# Incorporating phylogenetic information for the definition of floristic districts in hyperdiverse Amazon forests: Implications for conservation

**DOI:** 10.1002/ece3.3481

**Published:** 2017-10-16

**Authors:** Juan Ernesto Guevara Andino, Nigel C. A. Pitman, Hans ter Steege, Hugo Mogollón, Carlos Ceron, Walter Palacios, Nora Oleas, Paul V. A. Fine

**Affiliations:** ^1^ Department of Integrative Biology University of California Berkeley CA USA; ^2^ Yachay Tech School of Biological Sciences and Engineering Urcuqui Ecuador; ^3^ Keller Science Action Center The Field Museum Chicago IL USA; ^4^ Naturalis Biodiversity Center Leiden The Netherlands; ^5^ The Netherlands & Systems Ecology Free University Amsterdam The Netherlands; ^6^ Endangered Species Coalition Silver Spring Washington DC USA; ^7^ Universidad Central Escuela de Biología Herbario Alfredo Paredes Quito Ecuador; ^8^ Universidad Técnica del Norte Ibarra Ecuador; ^9^ Universidad Tecnológica Indoamérica Herbario UTI Quito Ecuador

**Keywords:** Amazon, Ecuador, endemism, phylogenetic beta diversity

## Abstract

Using complementary metrics to evaluate phylogenetic diversity can facilitate the delimitation of floristic units and conservation priority areas. In this study, we describe the spatial patterns of phylogenetic alpha and beta diversity, phylogenetic endemism, and evolutionary distinctiveness of the hyperdiverse Ecuador Amazon forests and define priority areas for conservation. We established a network of 62 one‐hectare plots in terra firme forests of Ecuadorian Amazon. In these plots, we tagged, collected, and identified every single adult tree with dbh ≥10 cm. These data were combined with a regional community phylogenetic tree to calculate different phylogenetic diversity (PD) metrics in order to create spatial models. We used Loess regression to estimate the spatial variation of taxonomic and phylogenetic beta diversity as well as phylogenetic endemism and evolutionary distinctiveness. We found evidence for the definition of three floristic districts in the Ecuadorian Amazon, supported by both taxonomic and phylogenetic diversity data. Areas with high levels of phylogenetic endemism and evolutionary distinctiveness in Ecuadorian Amazon forests are unprotected. Furthermore, these areas are severely threatened by proposed plans of oil and mining extraction at large scales and should be prioritized in conservation planning for this region.

## 1 INTRODUCTION

Ever since Wallace one of the main goals of biogeography has been the delimitation of biotic regions in order to circumscribe areas that are characterized not only by the same species pool but also potentially by the same evolutionary, geological–historical, and ecological processes. Thus, the spatial classification of biodiversity has strong implications for the understanding of the evolutionary and ecological processes underlying patterns of alpha and beta diversity (Kreft & Jetz, 2010; Li, Kraft, Yang, & Wang, 2015).

Located within the South America's Piedmonte del Napo region, the Ecuadorian Amazon has been recognized as one of the most biodiverse areas around the world (Bass et al., 2010; Funk, Caminer, & Ron, 2012; Myers, Mittermeier, Mittermeier, Da Fonseca, & Kent, 2000) and is especially famous for possessing the highest levels of tree and shrub diversity across the Amazon basin (Pitman et al., 2001; ter Steege et al., 2013, 2016; Valencia et al.,^2004^). Floristic inventories in the Ecuadorian Amazon have also been influential in our understanding of the concept of hyperdominance and patterns of relative abundance of species in the Amazon as well as floristic disruptions triggered by geology (Higgins et al., 2011; Pitman et al. 2008), suggesting that the assembly of the lowland Amazonian tree flora is the result of the interplay between edaphic specialization mediated by geological history and oligarchic tree communities. However, besides these efforts to determine both floristic and abundance patterns in Ecuador Amazon tree flora (Macía & Svenning, 2005; Pitman, Jorgensen, Williams, Leon‐Yanez, & Valencia, 2002; Pitman et al., 2001; Valencia et al., 2004), our understanding of the Ecuadorian Amazonian flora is quite limited due to significant geographic gaps in floristic assessments across the region. To date, the most complete floristic assessment of the Ecuadorian Amazon used both herbarium data and a one‐hectare plot network to delineate four floristic subregions (Guevara et al., 2016a). However, there has been no systematic attempt to define floristic regions using approaches that include both compositional and phylogenetic diversity, which is likely to provide additional insights to improve research‐based conservation policies Honorio.

In his pioneering work, Faith (1992) posited the concept of phylogenetic diversity as the sum of branch lengths of a phylogenetic tree along a minimum spanning path connecting the tips of the tree present in a location to its root. This measure has been the cornerstone of subsequent methods looking for the identification of regions of high‐phylogenetic endemism and/or evolutionary distinctiveness (Forest et al., 2007; Mishler et al., 2014; Redding & Moers, 2006; Rosauer, Laffan, Crisp, Donnellan, & Cook, 2009). Applied in a biogeographical‐conservation context PD provides a way to detect regions that contain assemblages of species that share the same evolutionary history and help us to elucidate the historical events that may have shaped these assemblages (Kraft, Baldwin, & Ackerly, 2010; Whittaker et al., 2005). Recent works have developed indexes such as Phylogenetic Endemism (WPE) defined as the sum of the branch lengths’ geographic range that a clade of the regional phylogenetic tree occupies in a particular region (Rosauer et al., 2009). Because phylogenetic endemism works as an analogy of weighted endemism described as a relative measure of endemism, we can use this index to better understand floristic changes across regions and simultaneously define conservation priority areas more effectively than using taxonomy alone (Laffan, Lubarsky, & Rosauer, 2010; Li et al., 2015).

Here, we present the results of an extensive one‐hectare plot network that represents the most comprehensive spatial sampling of the trees of the Ecuadorian Amazon to date in order to evaluate the patterns of floristic affinities in this hyperdiverse region and provide insights into conservation priorities from a phylogenetic context. In addition, we address the following questions: (i) What floristic classification of the Ecuadorian Amazon do our results support? (ii) To what extent are differences in species composition (taxonomic dissimilarity) across the region congruent with differences in phylogenetic composition (phylogenetic dissimilarity)? (iii) Are regions with high‐phylogenetic diversity (PD) areas with extraordinary evolutionary distinctiveness or endemism? (iv) Are areas characterized by high PD currently under formal conservation protection?

## 2 METHODS

### 2.1 Study area

**Figure 1:**
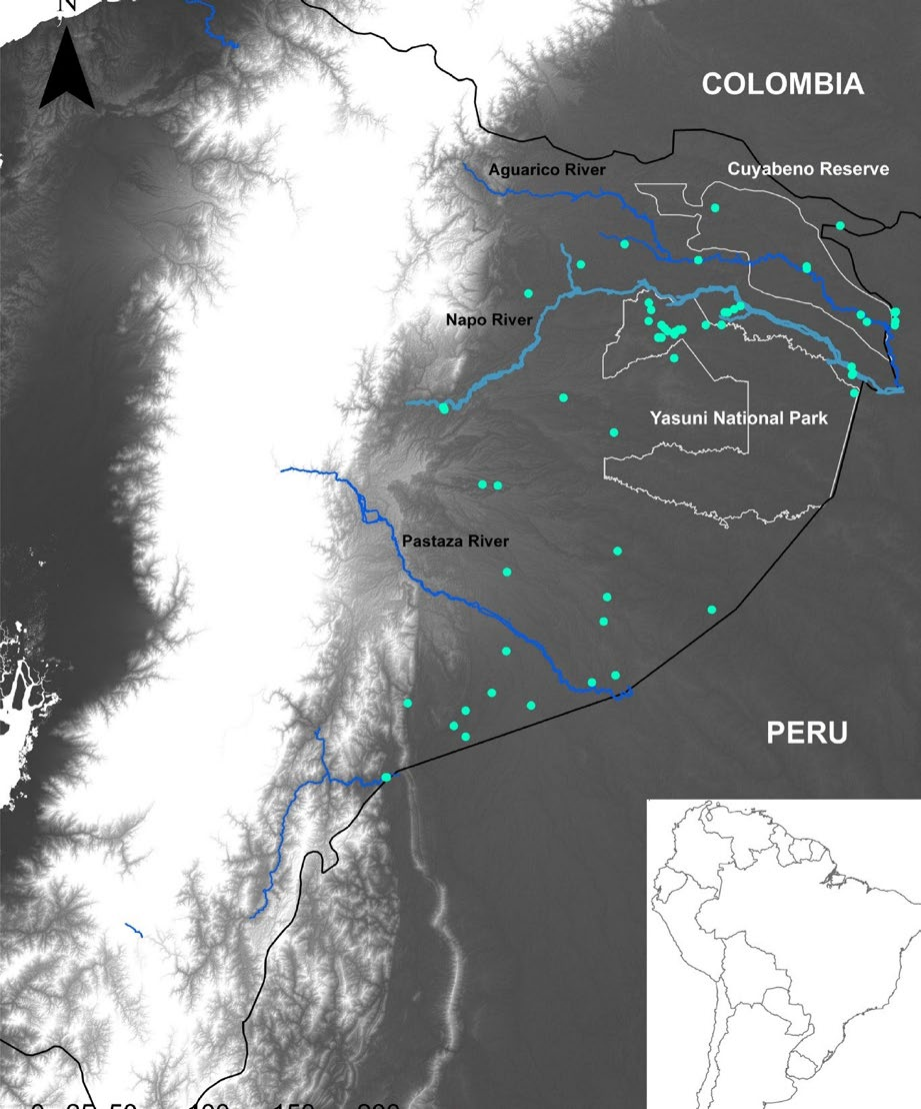
Map of locations of the 62 one‐hectare plots used in this study

Our study was carried out in the lowland Ecuadorian Amazon (Figure 1). We defined lowland Amazonia based on three parameters proposed as diagnostic factors for the definition of vegetation units for the Vegetation Map of Ecuador (Ministerio del Ambiente del Ecuador, 2013). The area includes two protected areas in the north, Yasuní National Park and Cuyabeno Reserve, whereas the southern portion of Ecuador Amazon contains no formal protected areas. Toward the northern portion of Yasuní National Park, the interfluvial landscape is mostly dominated by rolling hills interrupted by terrain depressions or *baixios* that vary in extent and levels of drainage (Pitman 2000). This landscape is interrupted by the Napo River that divides the most northern portion of the Ecuadorian Amazon from the rest. High and low terraces from Pleistocene origin dominate the northern and southern riverbanks of the Aguarico River, whereas the northern riverbank of the Napo River mainly consists of palm‐dominated swamps (Ministerio del Ambiente del Ecuador, 2013). The Pastaza River represents a geomorphological break in the landscape of Ecuador Amazon. South of this river the landscape is characterized by extensive plains of terra firme forests interspersed by swamps that are sometimes but not always dominated by palms. This area is known as the Pastaza fan which corresponds to a massive volcaniclastic alluvial fan deposited during the Holocene (Rasanen et al. 1987; Bernal et al. 2011). Finally, we sampled the lowland forests adjacent to the Cordillera del Condor, which is one of the areas of the Ecuadorian Amazon that remains most poorly explored in terms of floristic inventories. We sampled one plateau at 300–400 m on quarzitic sandstones (white sands) that represents the lowest altitude of Cordillera del Condor in Ecuadorian Amazon and also the first record of white‐sand habitats for the lowland Amazon of Ecuador (The correct citation is Ministerio del Ambiente del Ecuador, 2013.).

### 2.2 Tree community data

We established a network of 62 one‐hectare plots from 2000 to 2016 in the Ecuadorian Amazon including terra firme and white‐sand forests (Figure 1, Table 1). Our plot network includes many areas not previously visited by botanical researchers, namely the lower portion of Cordillera del Condor (five plots) and the Pastaza river watershed in Ecuador (10 plots). In each plot, we recorded, tagged, and identified all trees with diameter at breast height (dbh) ≥10 cm. Botanical collections for every tree species were collected, and duplicates were deposited and compared with botanical specimens from five herbaria (MO, QCNE, QCA, QAP, and F). Most of the new records and new species have been confirmed by taxonomic specialists from each group, but in other cases, our extensive experience in Amazonian tree species identification allows us to be confident about the accuracy of the taxonomy across the plot network. Finally, in order to perform phylogenetic and statistical analyses, we excluded unnamed morphospecies, which have been demonstrated to have weak effects on the detection of ecological patterns (Lennon, Koleff, Grenwood, & Gaston, 2001; Lennon, Koleff, Grenwoow, & Gaston, 2004; Pos et al., 2014).

**Table 1:** Results of multiple response permutation procedure and mantel tests for TBD and PBD

	*r* Mantel test	MRPP observed δ value	MRPP expected delta value	Within groups A statistic	*p*‐value
Phylogenetic beta diversity					
Phylogenetic beta diversity–taxonomic beta diversity	.912				<.001
Null phylogenetic beta diversity–taxonomic beta diversity	.445				<.001
Basal phylogenetic beta diversity–taxonomic beta diversity	.281				<.001
Definition of three floristic regions based on taxonomic beta diversity		0.754	0.8012	0.0645	.00009
Definition of three floristic regions based on phylogenetic beta diversity		0.4827	0.514	0.0611	.00009

### 2.3 Phylogenetic tree

We created a phylogenetic tree for 1,687 operational taxonomic units (OTUs) using as backbone the tree R20120829 (Li et al., 2015) from Phylomatic (Webb & Donoghue, 2005), which is based on the Angiosperm Phylogeny Group's system (APGIII, 2009). In order to assign branch lengths, we used the BLADJ algorithm in Phylocom (Webb, Ackerly, & Kembel, 2008) based on inferred nodes ages (Wikström, Savolainen, & Chase, 2001). Despite the fact that our regional phylogenetic tree is not fully resolved, recent studies have demonstrated that there is no significant difference between supertrees based on inferred node ages and trees using DNA in order to detect patterns at community or regional scale (Swenson, 2009).

### 2.4 Taxonomic and phylogenetic alpha diversity metrics

To estimate species diversity at each location/plot, we used Fisher's alpha index which calculates the number of species in a sample relative to the number of individuals therein based on the following formula:

$$S=\alpha \ln(1\;+\;\frac{n}{\alpha })$$

Where *S* is the number of species, FA is the Fisher's value per assemblage, and *N* is the number of individuals per plot. We used the Fisher's alpha index (α) based on two basic assumptions: The first one implies that tree species abundances usually follow a log series distribution and secondly the regional species pool is spatially homogeneous. Based on previous evidence, we can argue the first assumption is fulfilled (ter Steege et al., 2013), while the second assumption is still matter of debate but could be a good approximation for the Ecuadorian Amazon forests (Pitman et al.,^2002^). In addition, Fisher's alpha is a scale‐independent estimator that has a good discriminatory power to detect richness under the assumption that the number of species tends to infinity (Schulte et al. 2005).

In order to evaluate the standardized effect size of PD in each local community, we calculated the ses.mpd value for each plot using the independent swap algorithm as the null model (Gotelli, 2000) implemented in the “picante” package in R (Kembel et al., 2010). This metric measures the standardized effect of mean pairwise phylogenetic distance between communities. Positive values over a 1.96 confidence interval determine communities were mainly structured by more closely related species (phylogenetic clustering) than expected by chance, and negative values less than −1.96 confidence interval were communities assembled by more distantly related species than expected by chance (overdispersion) (Webb 2000)

### 2.5 Taxonomic and phylogenetic beta diversity

Investigating how phylogenetic relatedness among communities’ changes across environmental and spatial gradients allows us to make inferences about the different biogeographical histories of regional species pools with the strong analytical power of phylogenies (Graham & Fine, 2008). For instance, high levels of Taxonomic Beta Diversity can be congruent with high levels of Phylogenetic Beta Diversity if allopatric speciation by vicariance has promoted geographical separation of two areas for long periods of time, which in turn has led to long disparate evolutionary histories of communities in both areas. Conversely, high levels of TBD can be related to low PBD indicating that recent events of speciation via parapatry or sympatry may be the drivers of community assembly. We must also consider that species abundances might be correlated with phylogeny if traits associated to habitat specialization allow species of one or few clades to become abundant in a particular habitat or region. Abundance‐weighted phylogenetic metrics are essential to understand whether PD is concentrated in few dominant clades that would represent a great proportion of regional floras and therefore predictors of floristic breaks among regions.

TBD was calculated as the taxonomic dissimilarity between pairs of local communities (1‐Sorenson index), whereas PBD was calculated with the Phylo Sorenson index as a measure of the degree of phylogenetic relatedness between pairs of local communities. In order to be consistent with the metrics used to evaluate taxonomic beta diversity, we used the complement of the Phylo Sorenson index to establish a phylogenetic dissimilarity metric (1‐Phylo Sorenson) (Bryant et al., 2008; Graham, Parra, Rahbeck, & Mcguire, 2009).

In order to test whether TBD is a good predictor of PBD, we compared the observed and expected values of PBD. In order to do this, we calculated the expected values of PBD based on a null model that makes random draws from the regional species pool (here defined as the total number of species in our plot network). This null model randomizes the community data matrix with the independent swap algorithm developed by Gotelli (2000), maintaining species occurrence frequency and sample species richness. Thus, if the observed values of PBD are less than the expected values based on the null model, we infer that pairs of compared communities are composed of lineages that are closely related. Conversely, if values of PBD are greater than expected based on the null model, then pairs of communities are composed of lineages that include distant relatives. Both mantel tests and multiresponse permutation procedure were performed to test the significance of the correlation between patterns of taxonomic beta diversity and phylogenetic beta diversity as well as the significance of the difference between groups of sites based on permutation tests of among‐ and within‐group dissimilarities (Legendre & Legendre, 2012; Mielke, 1991).

### 2.6 Ordination

Meta Nonmetric Multidimensional Scaling (NMDS) with both taxonomic and phylogenetic dissimilarity matrices was performed in order to have a graphical depiction of the floristic relationships of the 62 one‐hectare plots in the Ecuador Amazon basin. We used the first two dimensions in the ordination and 1,000 random starting iterations in order to obtain the lowest stress value that determines the best solution for that ordination. In order to delineate floristic units based on dissimilarity, we used the two‐first axes of the NMDS ordination based on both Sorenson and Phylosorenson indexes. Therefore, instead of showing the ranked values of the original dissimilarity matrix in a two‐dimensional space, we show raw values of phylogenetic and taxonomic dissimilarity for both axes.

### 2.7 Phylogenetic endemism, evolutionary distinctiveness, and imbalance of abundance at clade level

Finally, we calculated the weighted phylogenetic endemism, Abundance‐weighted evolutionary distinctiveness (AED) and imbalance at clade level (IAC) following the algorithms developed by Rosauer et al. (2009) and Cadotte et al. (2010), respectively. Weighted phylogenetic endemism (WPE) is defined as the sum of branch lengths divided by the clade range for each branch on the spanning path linking a set of taxa to the root of the tree (Rosauer et al., 2009). AED measures the evolutionary distinctiveness of species based on abundance and phylogenetic distances according to the following formula:

$$\text{AED}=\sum_{e\in s(T,i,r)}\frac{\lambda_{e}}{n_{e}}$$

Therefore, AED is not just proportional to the phylogenetic distances but also to the distribution of individuals in a particular *e* edge of length *k* in the set *s* (*T,i,r*) that connects species *i* to the root, *r* and *S*_*e*_ are the number of species that descend from edge *e*.

Finally, the IAC index measures the relative deviation in the abundances distribution of individuals in any clade based on the null expectation that individuals are evenly partitioned between clade splits (Cadotte et al., 2010).

$$\text{IAC}=\frac{\sum_{i\;=\;1}^{S}n_{i}-{}^{\wedge }n_{i}}{v}$$

Where *n*_i_ is the number of lineages originating at node *k* of *v* nodes in the set *s*(*T,k,r*). This is the number of nodes between node *k* and the *r* root in the tree *T,* meanwhile ^*n*_*i*_ is the expected abundance of species *i*.

### 2.8 Spatial models with taxonomic and phylogenetic diversity metrics

Several software packages for the spatial analysis of biodiversity have been developed in the past 10 years (e.g., Biodiverse, GDM) (Ferrier, Manion, Elith, & Richardson, 2007; Laffan et al., 2010), radically changing and improving our understanding of the spatial distribution of both taxonomic and phylogenetic diversity. The great majority of these analyses use a moving window approach that predefine a window around a group (e.g., site collection, plots) in a dataset to then calculate appropriate statistics for each group based on the neighborhoods that fall within such window (Laffan et al., 2010). However, as a caveat one must consider that when there is not complete spatial coverage within a region there is no way to predict values of taxonomic and phylogenetic turnover across space. Therefore, we used a different approach to predict the spatial variation of both taxonomic and phylogenetic beta diversity and abundance‐based metrics for taxonomic and phylogenetic diversity. In order to perform this analysis, we divided the Ecuadorian Amazon into 0.5 degree grid cells (55 × 55 km) which is a spatial scale that allows us to have a balance between accuracy and detail when performing the spatial analysis (Kreft & Jetz, 2010; Keil et al. 2012). It has been demonstrated that grain size affects beta diversity estimations and that increasing grain size should produce lower beta diversity in high species richness areas (Lennon et al., 2001; Keil et al. 2012). This is mainly determined by the fact that there is an intrinsic relationship between the SAR and species turnover. In other words by increasing the grain size, there is less room for variation in species composition because more of the regional species pool is being accounted for (Lennon et al., 2001). On the other hand by reducing the grain size, we would increase the number of grid cells containing plots in contrasting habitats (terra firme vs. white sands) therefore overestimating the predicted values of both beta and phylogenetic beta diversity (Keil et al. 2012). In addition, because finer grain size could lead us to increase the sampling bias introduced by the nonuniform distribution of plots, intermediate grid cell size may avoid underestimation of phylogenetic and taxonomic beta diversity values. Moreover, while there is some level of uncertainty in the interpolation of phylogenetic metrics of unsampled or under sampled areas, we argue this may not affect the patterns we found. In fact the gra*i*n size we defined to perform our spatial analysis has been demonstrated to be appropriate to not under or overestimate predicted values of dissimilarity. In addition, because broader or finer grain size could lead us to increase the sampling bias introduced by the nonuniform distribution of plots, intermediate grid cell size may avoid underestimation of phylogenetic and taxonomic beta diversity values (Kreft & Jetz, 2010, Keil et al. 2012).

In order to avoid these bias and because our data are not presence–absence records of each grid cell we calculated the mean values of both PBD and TBD for each plot with respect any other in the plot network. Then we used these average values to perform interpolation across the region. A Loess spatial regression model was used to predict both taxonomic and phylogenetic turnover. To obtain the most accurate fit, we used default parameters for our Loess regression: a 0.75 span was used to find the best smoothing average, and a degree 2 polynomial was set to reduce variance. We used this method due to its inherent flexibility compared with other interpolation techniques. Because our data are irregularly distributed, Loess interpolation allows us to fit at the local scale individual values of taxonomic and phylogenetic diversity across space using the average of each of these values at location *x* with grid cells in the neighborhood of *x*. In order to perform this, the Loess method sets the size of the neighborhood with respect to location *x* with the parameter α. All the analyses were performed with the packages picante (Kembel et al., 2010), vegan (Oksanen et al.,^2015^) and using custom functions on the R platform.

## 3 RESULTS

### 3.1 Alpha diversity patterns

The highest Fisher's alpha values were found in a cluster of plots at the intersection of a latitudinal band between .5 and .8 degrees and a longitudinal band between 76 and 76.5 degrees (Fig. S1). This peak of taxonomic diversity is congruent with peaks of phylogenetic alpha diversity across the region (Fig. S1, Table 1).

### 3.2 Floristic affinities in the Ecuadorian Amazon

**Figure 2:**
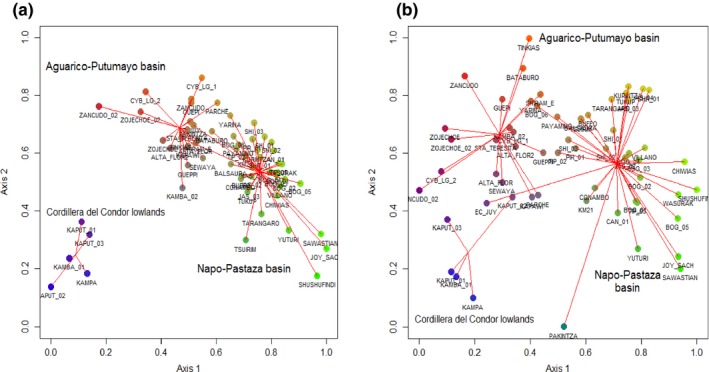
Nonmetric multidimensional ordinations based on the (a) phylogenetic dissimilarity and (b) taxonomic dissimilarity for 62 one‐hectare plot network in terra firme forests of Ecuador Amazon. (a) Phylogenetic dissimilarity‐based NMDS ordination defines three floristically distinct districts; the Aguarico‐Putumayo district (green palette dots), the Napo‐Pastaza district (orange palette dots), and the Cordillera del Condor lowlands (blue palette dots). (b) Taxonomic dissimilarity‐based NMDS ordination defines three floristically distinct districts; the Aguarico‐Putumayo district (orange palette dots), the Napo‐Pastaza district (green palette dots), and the Cordillera del Condor lowlands (blue palette dots). RGB colors represent dissimilarity values plotted in the two dimensional space of the ordination. Spiders diagram represents associated groups of plots; sites are connected to the centroid of each class, in this case, a floristic region defined on the basis of the results of the mrpp analysis based on the geographic location of each plot

**Figure 3:**
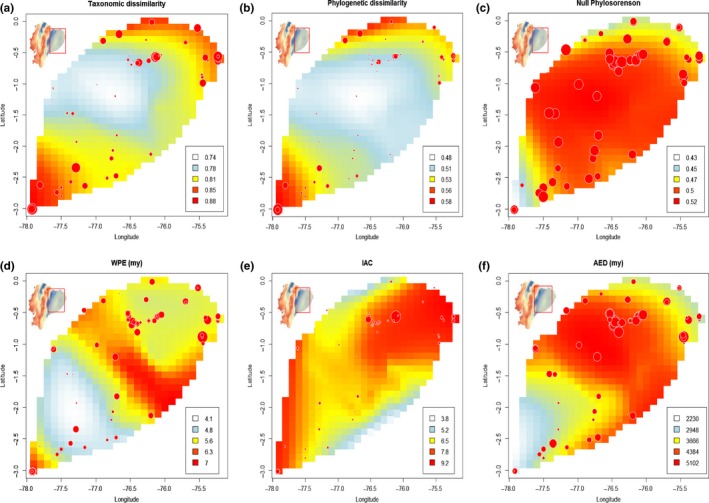
Spatial variation of different measures of phylogenetic diversity and phylogenetic beta diversity across Ecuador Amazon. Spatial interpolation was based on Loess regression with 0.5 degree grid cell span. (a) Taxonomic beta diversity measured as 1‐Sorenson as a proxy of taxonomic dissimilarity. (b) Phylogenetic beta diversity measured as 1‐Phylosorenson as a proxy of phylogenetic dissimilarity. (c) Null phylogenetic beta diversity measured as 1‐Phylosorenson based on 1000 randomized matrices using swap algorithm. (d) Weighted Phylogenetic endemism. (e) Imbalance of Abundances at clade level and (f) Abundance‐weighted evolutionary distinctiveness. Red and orange colors represent higher values for each metric, while lighter yellow and light blue colors represent lower values for each metric. The size of the dots is ranked according each metric, so lower values are presented by small sized dots and higher values for each metric correspond to larger dots

Taxonomic‐based NMDS analysis (stress function 0.1048091) led to the definition of three floristic districts (Figure 3b), and the MRPP analysis. A similar pattern was found with the phylogenetic‐based nonmetric multidimensional analysis (NMDS) (stress function 0.1019252). These regions correspond to the forests located in the interfluvial areas between Aguarico‐Putumayo basin (Aguarico‐Putumayo basin), the interfluvial areas between the Napo and Pastaza rivers and the Cordillera del Condor lowlands (Figures 1 and 2A).

MRPP analysis based on Phylosorenson values support the delimitation of three floristically distinct units as shown by the delta values (Table 1). Thus, there is highly significant difference between groups of sites according to the biogeographical subdivision supporting the delimitation of three floristic subregions in Ecuador Amazon (Table 1).

### 3.3 Beta diversity patterns

The spatial distribution of taxonomic and phylogenetic beta diversity was very similar. We found a tight correlation between TBD and PBD (*r* = .9043, *p* ≤ .001), which indicates that phylogenetic dissimilarity can be predicted by taxonomy (Table 1, Fig. S2). Nevertheless, we found a weaker correlation between taxonomy and phylogeny when the standardized ses.mpd index was included in analysis (*r* = .3016, *p* = .002). When comparing the observed values of phylogenetic turnover against the expected values based on our null model we found lower observed phylogenetic turnover than expected (Fig. S2).

### 3.4 Evolutionary distinctiveness and phylogenetic endemism

High WPE values were concentrated in areas such as Condor Cordillera lowlands and the Aguarico‐Putumayo basin, whereas the lowest values were concentrated in the southern portion of the Napo–Pastaza basin. The spatial distribution of WPE showed that some areas to the southeast of the Ecuadorian Amazon basin are predicted to represent areas with high‐phylogenetic endemism. In general, high AED values were concentrated in areas such as Napo–Pastaza basin and the most northwestern part of this region (Figure 3F). The spatial distribution of AED shows that a great portion of the southern Ecuadorian Amazon is characterized by moderate to high levels of evolutionary distinctiveness. A different pattern arises when the spatial distribution of AED is considered, with low‐AED values concentrated in areas that correspond to Cordillera del Condor region (Figure 3F).

We also found significant differences in the spatial distribution of Imbalance of Abundances at Clade level (IAC) (Figure 3E). This is confirmed with the spatial distribution of abundances across the Ecuadorian Amazon, as there is a disproportionate dominance of clades such as Arecaceae, Moraceae, Fabaceae, or Myristicaceae in areas of the Napo–Pastaza basin. We also found higher than predicted IAC values in regions that correspond to the lowland of Cordillera del Condor and some areas of the Pastaza fan (Figure 3E).

## 4 DISCUSSION

### 4.1 Floristic patterns

Our results improve a previous classification of the floristic relationships in Ecuadorian Amazon (Guevara et al. 2016), which delimited four floristic regions. We argue that previous regionalization was made on the basis of arbitrary boundaries to delimitate distinct floristic units without any statistical support. The main difference is the strong floristic affinities between the previously separated Pastaza basin and Napo‐Curaray basin. While our ordination does show some degree of overlapping between the Napo–Pastaza and the Aguarico‐Putumayo basins, we argue that the differences between the mean dissimilarity for each group centroid are enough to consider them as different floristic units. This is confirmed with the results of the multiresponse permutation procedure (Table 1). Because, this method allows us to deal with increasing community heterogeneity and also can help to correct the loss of sensitivity due to this fact we argue our results address properly the inherent high variation in species composition between sample units (plots).

Some groups such as *Inga, Ocotea, Pouteria, Virola, Eugenia,* and *Calyptranthes* are species‐rich genera that exhibit peaks of diversity in Yasuní National Park. The spatial distribution of phylogenetic beta diversity (phylogenetic dissimilarity) shows stronger patterns than expected from the null model in the most southern and northern portion of Ecuadorian Amazonia, which is congruent with the delineation of the Condor Cordillera lowlands and the Aguarico‐Putumayo as distinct floristic districts. Some elements from regions with biogeographic affinities with the Guiana Shield have been recorded only in the northern portion of the Aguarico‐Putumayo basin, and these unusual trees include genera such as *Sterigmapetalum, Chaunochiton, Neoptychocarpus Macoubea, Podocalyx, Pogonophora, Bothryarrena, Clathrotropis, Ruizterania,* and *Neocalyptrocalyx*. Almost 90% of the new records in this district include species that are locally abundant in areas of the Middle Caquetá in the Colombian Amazon and in areas near Manaus, Brazil (De Oliveira and Daly 1999; De Oliveira and Mori 1999; Duque, Sánchez, Cavelier, & Duivenvoorden, 2002; Pitman et al., 2003). Thus, we think the northeastern portion of the Ecuadorian Amazon may represent the westernmost edge of Amazon with floristic influences of Central Amazonia and the Guiana Shield region and might be defined as a transitional area between these regions and the westernmost portion of the Ecuadorian Amazon that has more of an Andean floristics influence. We propose that this floristic influence may include areas of Colombian and Peruvian Amazon along a west–east axis but toward the north bank of the Napo River. This is consistent with earlier studies that have posited that a strong floristic disruption between forests located to the west and those located to the east of the Ecuador‐Peru border might also represent a shift in geological formations from nutrient‐rich Miocene to nutrient‐poor Pleistocene‐based sediments (Higgins et al., 2011; Pitman et al. 2008). Moreover, we found support for this hypothesis in a preliminary analysis comparing our plot network with a set of plots in Peruvian Amazon with strong floristic affinities with Middle Caquetá region (Pitman et al. 2008; Fig. S3). Our Loess regression model predicts high spatial turnover of lineages in the lowland forests (<500 masl) adjacent to the Cordillera del Condor district (Figure 3B.). This region is one of the most floristically distinct areas within Ecuadorian Amazon (Figure 2). The confluence of several floras, including widely distributed elements of the Amazon piedmont, the flora of Guyana Shield tepuis and the region of Iquitos, Peru on mixed soils determine the patterns we found in this region. Some taxa that are predominant in this area include the genera *Centronia*,* Pachira*,* Micrandra*,* Diclinanonna*,* Parkia*,* Aspidosperma,* and *Sterigmapetalum* (Appendix S1).

### 4.2 Can PBD be predicted by TBD?

Our results highlight the benefits of the use of complementary phylogenetic methods to determine strong turnover in floristic composition and also their importance for conservation purposes. We found that the observed levels of lineages turnover (PBD) are significantly lower than expected. A similar pattern has been found in two regional analyses of North American Angiosperms and white‐sand forests across the Amazon basin (Guevara et al., 2016a,^2016^; Qian, Swenson, & Zhan, 2013). Lower PBD than TBD may be the result of the spatial turnover of species that are nested in similar clades which in turn leads to floras mainly composed of the same phylogenetic components. Our results support the hypothesis that PBD can be predicted by TBD, and lower PBD than expected based on null TBD may be suggestive of recent divergence across strong environmental gradients or biogeographic boundaries promoting speciation for subsets of regional species pool (Graham et al., 2009). Moreover, the predicted spatial distribution of PBD not only represents spatial variability in lineage composition but should also represent variability in the set of traits for subsets of the regional species pool. This suggests a potential scenario in which parapatric speciation might be a general process shaping Amazon forest composition. Nonetheless, current evidence suggests that allopatric speciation after dispersal might be a major evolutionary driver of speciation in Amazon tree lineages (Dexter et al., 2017). Therefore, it will be important to carry out subsequent research at clades levels to elucidate whether these can be considered general mechanisms for the formation of species pool in Amazonian forests (Fine & Baraloto, 2016).

### 4.3 Are regions with high levels of PD areas with high levels of evolutionary distinctiveness and endemism?

The spatial distribution of WPE and PBD determined that communities located in Cordillera del Condor lowlands may be characterized by high levels of WPE and PBD meaning that there is a high replacement of lineages with short‐geographic ranges compared with communities in the other floristic districts of Ecuador Amazon (Figure 3B,D). High levels of WPE can be explained by the presence of white‐sand specialist taxa recently diverged from adjacent terra firme sister clades (Fine et al. 2013; Misiewicz & Fine, 2014). Low levels of AED are also congruent with this scenario because individuals corresponding to species and clades sharing low evolutionary distinctiveness may be dominant in this region (Figure 3F). Simultaneously, the spatial distribution of IAC and AED determined that communities in the Napo‐Pastaza watershed exhibit the highest IAC values meaning that there is significant phylogenetic imbalance in the distribution of abundance and certain unique clades dominate this area. Moreover, because this region is also characterized by high AED values one might argue that the most abundant species and clades are sharing disproportionately long‐branch lengths corresponding to common species from clades that have extremely ancient divergent times from one another. The abundance and diversity of Magnoliids, Arecaceae, and Moraceae, which are remarkably dominant in this region, might explain this pattern. Moreover, genera such as *Ocotea, Virola, Otoba,* and the monotypic palm genus *Iriartea* exhibit peaks of abundance in areas like Yasuní National Park.

Conversely, low values of AED in areas corresponding to Condor Cordillera lowlands and the adjacent forest of Pastaza fan watershed are consistent with the hypothesis that the composition of these forests is characterized by turnover of recently diverged lineages. We found that taxa dominant in the white‐sand forest of the surroundings of Iquitos and the upper Morona river watershed and taxa which are also dominant in medium elevation plateaus of El Condor Cordillera are important floristic components of the regional flora of Cordillera del Condor lowlands (Fine, Garcia Villacorta, Pitman, Mesones, & Kembel, 2010). Some potential mechanisms appear to be responsible of the pattern we found, parapatric speciation across gradients of soils might trigger speciation if divergent selection promotes adaptations to different extremes of a soil gradient (Fine et al., 2013). This process could occur more rapidly than in allopatric populations if the differences in soils are extreme enough to inhibit gene flow across soil boundaries (Coyne and Orr 2004).

### 4.4 Implications for conservation

The inclusion of an evolutionary approach in any analysis of beta diversity can contribute significantly to scientific research‐based conservation policies. Because species‐centric conservation research solely takes into consideration a snapshot of the fractal nature of the tree of life without including phylogenetic data we miss all the information that genealogical relationships between organisms can give us. Currently, many conservation priority‐setting exercises tend to be solely focused on species‐level data and have proved to be a poor predictor of both species richness and threatened species identification (Orme et al., 2005). We found that despite a high correlation between species richness and PD, the predicted spatial distribution that incorporates phylogenetic information shows critical new details. For example, areas that currently are unprotected and exhibit high Fisher's alpha values are also areas with relative high PD. These areas include the lowlands of Cordillera del Condor and the Pastaza fan watershed.

Regarding the predicted spatial patterns of PBD, we found that areas with high replacement of lineages could be considered as important priorities for conservation purposes because so many phylogenetically distant lineages coexist across the landscape. In the Ecuadorian Amazon, the subregions with the highest values of phylogenetic turnover correspond to areas that include national parks (e.g., Cuyabeno reserve in the Aguarico‐Putumayo‐Caquetá district) but also areas that are under some level of threat. For example, the lowlands of Condor Cordillera region and the Pastaza basin are regions threatened by massive plans for new hydroelectric dams, large‐scale gold mining projects, and oil extraction (Fine et al., 2013; Finer, Jenkins, Pimm, Keane, & Ross, 2008).

The spatial distribution of AED and WPE showed contrasting patterns with areas of the Ecuadorian Amazon that have no formal protection characterized by high levels of WEP and low levels of AED. Areas such as the Cordillera del Condor lowlands represent areas with low evolutionary distinctiveness meaning that the loss of species due to deforestation, mining or infrastructure development would represent a loss of unique lineages that have recently evolved. Furthermore, this loss would be related to the loss of lineages with restricted geographic ranges and that represent short branches of the regional phylogenetic tree. While we acknowledge that priority conservation areas have been largely defined based on high evolutionary distinctiveness (Cadote & Davies 2010; Jetz et al. 2014), we argue that areas representing lower values of phylogenetic distinctiveness should be considered priority areas for conservation if high‐phylogenetic endemism and high‐lineages turnover are also present in the same area. This means that lineages recently diverged from ancestors with restricted geographic ranges are more prone to suffer extinction by shrinking populations if these lineages have not had enough time to evolve adaptations to shifts in environmental conditions (Sandel et al., 2011). Because changes in climate have been correlated with high‐extinction risk in several taxonomic groups, we argue that this phenomenon could lead to high‐extinction levels in the southernmost part of the Ecuadorian Amazonia.

Most of the evolutionary lineages contained in the regional species pool of Ecuador Amazon are currently harbored within National Parks. However, there are some caveats to consider. For example, 45% of Yasuní National Park, located in the Napo‐Tigre watershed, overlaps with existing oil concessions, and meanwhile, 22% of the Cuyabeno Reserve in the Aguarico‐Putumayo‐district is currently also open for oil concessions (Lessmann, Fajardo, Munoz, & Bonaccorso, 2016). Even more alarming is the fact that 19 of the 25 ecosystems of lowland Ecuador Amazon are found within areas that are open for oil exploration, particularly toward the southern portion of the Pastaza fan watershed and Cordillera del Condor lowlands where Ecuador's greatest amount of evolutionary distinctiveness and phylogenetic endemism is concentrated.

Recently, Lessmann, Munoz, and Bonaccorso (2014) assigned a low‐to‐medium range in conservation priority to areas that correspond to the southern floristic districts we described here as regions containing both unique and geographically restricted evolutionary information. The approach used by these authors to define conservation priorities areas included richness maps based on species distribution models and maps of environmental vulnerability. However, we think that our results represent significant improvements upon these models. Here, we have demonstrated that areas with low AED values could be assigned as areas of mid‐to‐high levels of priority in a conservation context if the same areas exhibit high values of WPE, PBD. Moreover, we have shown that areas characterized by the dominance of recently diverged lineages with restricted ranges correspond to floristically unique units located toward the south of the Ecuadorian Amazon. Our finding that the turnover in species composition in areas with high endemism is due to species with low‐phylogenetic distinctiveness, suggests that recent speciation has led to high‐beta diversity. This is consistent with a model by which speciation processes are highly dynamic and correspond to the evolution of habitat diversity and/or climate changes during the Pleistocene, in the last 2 million of years. We argue that conservation of these areas is particularly critical in order to maximize the preservation of the evolutionary processes that underlie the origin of Ecuador's extraordinarily high tree diversity. This highlights the necessity to develop new conservation plans for this region taking into account the current and potential pervasive negative effects of mining, dam construction, and oil extraction.

## AUTHOR CONTRIBUTIONS

J.E.G. conceived the idea, analyzed data, and wrote the manuscript, P.V.A.F. reviewed and contributed to the writing of the manuscript, H.T.S. contributed with scripts in R to analyze data. All authors read and approved the final version of the manuscript.

## CONFLICT OF INTEREST

None declared.

## Supporting information


** **

** **
