# Supplementary material for: Incorporating phylogenetic information for the definition of floristic districts in hyperdiverse Amazon forests: Implications for conservation
Source: Ecol Evol. 2017 Oct 16;7(22):9639–50. doi: 10.1002/ece3.3481 (PMC5696432; doi:10.1002/ece3.3481)
Supplement: Supplementary file 2 [file ECE3-7-9639-s002.docx]

*Ecology and Evolution*

**Incorporating phylogenetic information for the definition of floristic districts in hyper-diverse Amazon forests: implications for conservation**

Juan Ernesto Guevara^1,2^, Nigel C.A. Pitman^3^, Hans ter Steege^4^, Hugo Mogollón^5^, Carlos Ceron^6^, Walter Palacios^7^, Nora Oleas^8^, Paul V.A. Fine^1^

^1^ Department of Integrative Biology, University of California, Berkeley, CA 94720-3140, US

^2^ Yachay Tech, School of Biological Sciences and Engineering, Urcuqui, Ecuador

^3^ Keller Science Action Center, The Field Museum, 1400 South Lake Shore Dr., Chicago, IL 60605-2496 USA

^4^Naturalis Biodiversity Center, Vondellaan 55, Postbus 9517, 2300 RA Leiden, The Netherlands & Systems Ecology, Free University, De Boelelaan 1087, Amsterdam, 1081 HV, The Netherlands

^5^ Endangered Species Coalition, Silver Spring, United States of America

^6^ Universidad Central, Escuela de Biología Herbario Alfredo Paredes, Quito, Ecuador

^7^Universidad Técnica del Norte, Ibarra, Ecuador

^8^ Universidad Tecnológica Indoamérica, Herbario UTI, Quito, Ecuador

**Clustering algorithms**

In order to obtain the cluster analysis that best represent the floristics affinities of the tree communities we studied we decided to use seven well known hierarchical cluster algorithms. These methods include Unweighted Pair-Group Arithmetic Average (UPGMA), the Weighted Pair-Group Arithmetic Average (WPGMA) or McQuitty method, Single Linkage, Complete Linkage, Ward, the median and centroid.

In order to compare the results of the best cluster algorithm based on conventional hierarchical clustering methods we decide to compare these results with a unbiased method of regionalization recently developed by Daporto *et al.* (2015). We decided to use the recluster.region function from the package recluster (Daporto *et al.* 2013) due to its sensitiveness to small to mid-spatial scales analyses. This function produces n trees by randomly re-ordering the original row order of the dissimilarity matrix and then the trees are trimmed to different nodes producing an increasing number of clusters. A final hierarchical clustering is applied generating an interval of maximum and minimum number of clusters and a consensus tree is generated. The results are summarized in a matrix providing number of clusters for each solution (k), the associated mean number of clusters obtained by node cuts (clust), the silhouette (silh) value and the explained dissimilarity (ex.diss). Silhouette values range between -1 and +1, with a negative value indicating that most cells/plots are probably located in an incorrect cluster. These values measures the strength of the partition of objects (plots/cells) by comparing the minimum distance between a particular plot and the most similar plot belonging to any other cluster and the mean distance of that plot with other belonging to the same cluster (Daporto  *et al*. 2015). Then the we used the function recluster.boot for bootstrapping of nodes in the original consensus tree using 1000 consensus trees to find the final consensus tree.

All the analyses were carried out with the packages recluster (Daporto *et al.* 2015) and the package vegan (Oksanen *et al.* 2016) from the R platform.

**Literature cited**

Daporto, L., Ramazzotti, M., Fattorini, S., Talavera, G., Vila, R. & Dennis, R.L.H. (2013) recluster: an unbiased clustering procedure for beta-diversity turnover. *Ecography,* **36,** 1070–1075.

Daporto, L., Ciolli, G., Dennis, R.L.H, Fox, R. & Shreeve, T.G. (2015) A new procedure for extrapolating turnover regionalization atmid-small spatial scales, tested on British butterflies. *Methods in Ecology and Evolution,* **6**, 1287–1297.

Oksanen, J., Blanchet, F.G., Kindt, R., Legendre, P., Minchin, P.R., O'Hara, R.B., Simpson, G.L., Solymos, P., Stevens, H.M.H & Wagner, H. (2015) vegan: Community Ecology Package. R package version 2.3-0.

**Supporting Information**

Appendix S1. Indicator Species Analysis for 80 one plot network established in Ecuador Amazon forests (APC= Aguarico-Putumayo-Caqueta basin, CCL = Cordillera del Condor lowlands, NP= Napo-Pastaza basin, PF= Pastaza fan basin). Bold species names represent taxa significantly associated (p ≤ 0.05) to one or more regions based on the following attributes: IV$maxcls = Region in which species has maximum indicator value, IV$indcls = Indicator value, IV$pval = the probability of obtaining the highest indicator value based on 1000 iterations.

**Supplementary figures legends**

Figure S1. Comparison between Taxonomic Diversity (measured as Fisher’s alpha index) and Phylogenetic diversity (measured as ses.mpd index) in Ecuador Amazon. Black lines represent the best fit for the relationship between latitude-longitude vs. Fisher’s alpha and Rao’s index based on loess interpolation.

Figure S2. Correlation between PBD and TBD measured as 1- Phylosorenson and 1- Sorenson respectively. Blue dots represents observed values of PBD while gray dots represents null values of PBD based on 1000 randomizations of presence-absence matrix (sites by species) using swap algorithm. A) Results for the regional species phylogeny based on the Phylomatic backbone tree; B) Results for the regional species phylogeny of 480 tree species based on the markers atpB, matK, ndhF, psbBTNH, rbcL, rpoC2, rps16, and rps4.

Figure S3. Non-metric multidimensional ordinations based on the taxonomic dissimilarity and b) phylogenetic dissimilarity for 130 one hectare plot network in terra firme Amazon forests of Ecuador, Peru, Colombia and Brazil. The ordination defines floristically affinities between plots located in the most northwestern portion of Ecuador Amazon and Cordillera del Condor lowlands with plots located in Putumayo-Caquetá and some Central Amazon plot with strong influence of Guiana Shield flora. RGB colors represent dissimilarity values plotted in the two dimensional space of the ordination. Spiders diagram represents associated groups of plots; sites are connected to the centroid of each class, in this case a floristic region defined on the basis of the results of the ordination. Ellipses represent the 95% confidence interval in grouping plots as part of a particular group of similar floristic units.

Figure 1.


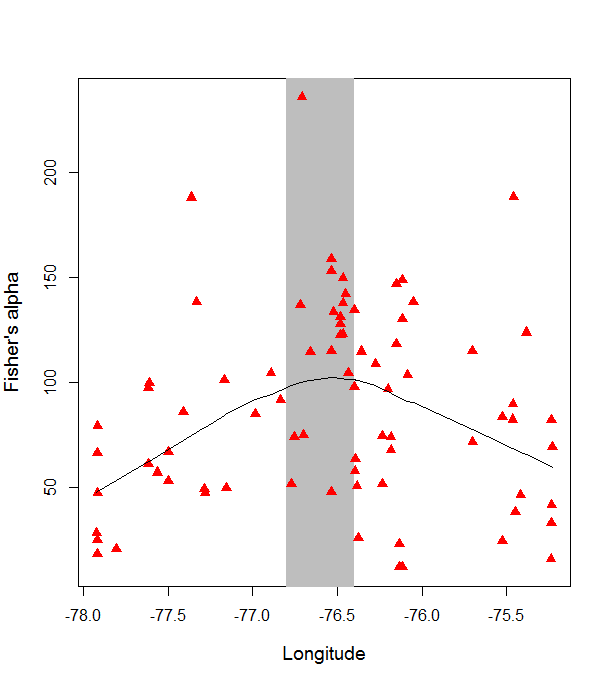


**(A)**


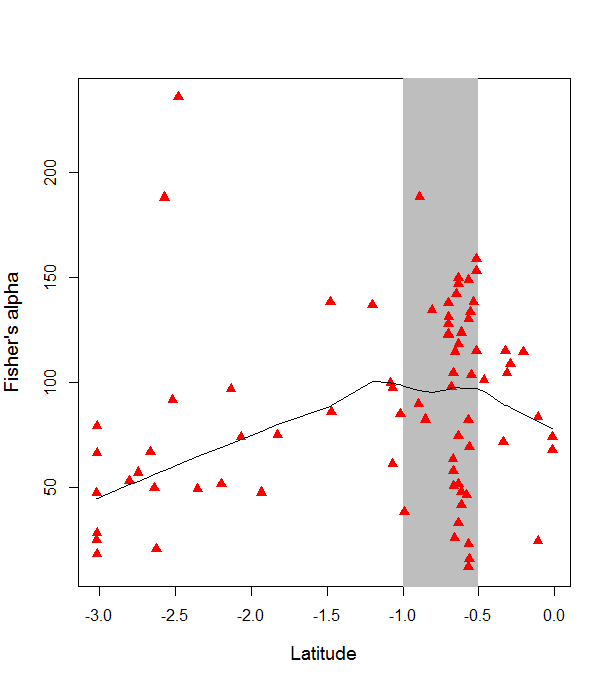

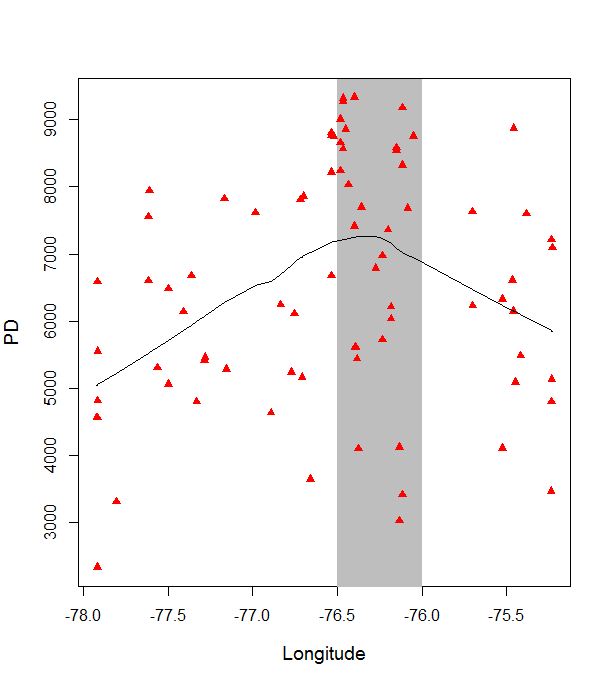


**(C)**


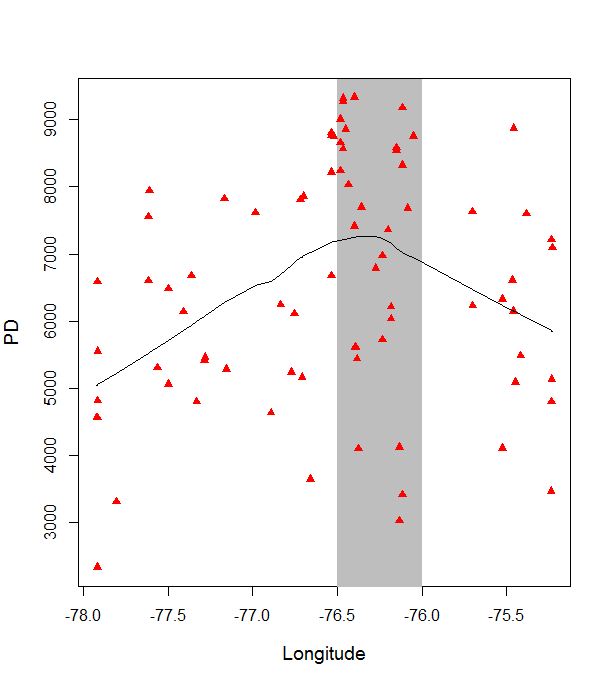


**(D)**

**(B)**

Figure 2.


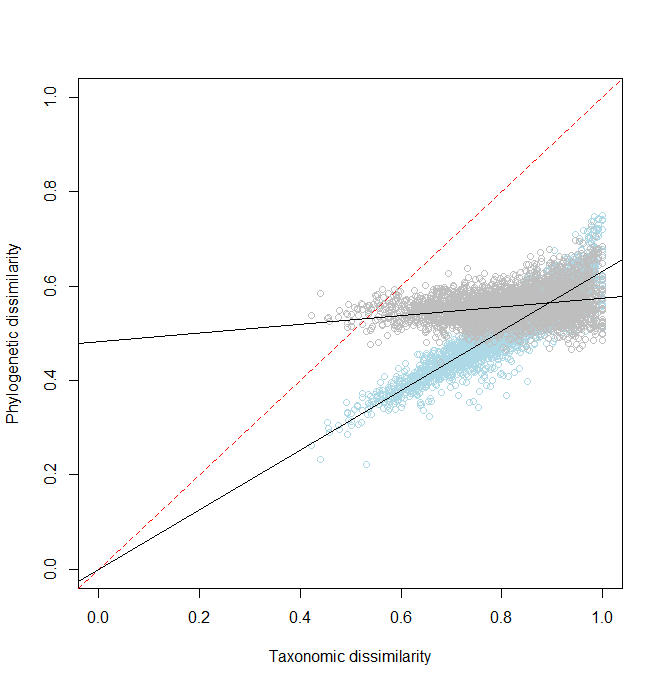

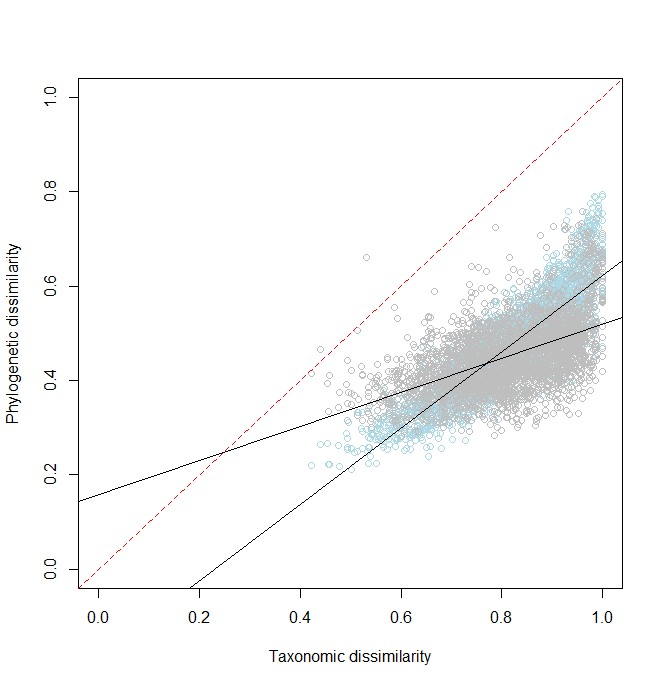


Observed

Expected

**(A)**

**(B)**

Figure 3.


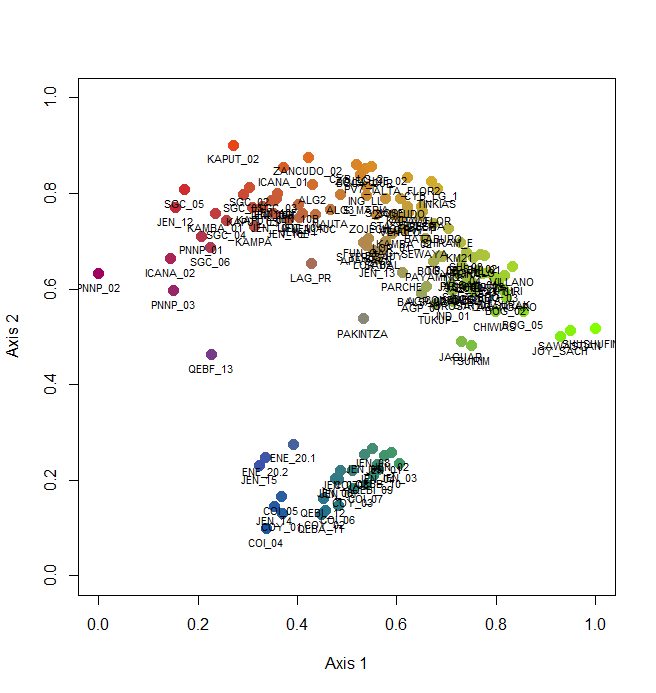


**Ecuadorian Amazon plots**

**Napo-Putumayo-Caquetá basin (Ecuador –Peru-Colombia)**

**Flooded forests (Peru-Brazil)**
